# A Cross-Sectional Examination of the Mental Wellbeing, Coping and Quality of Working Life in Health and Social Care Workers in the UK at Two Time Points of the COVID-19 Pandemic

**DOI:** 10.3390/epidemiologia2030017

**Published:** 2021-06-22

**Authors:** Paula McFadden, Ruth D. Neill, John Moriarty, Patricia Gillen, John Mallett, Jill Manthorpe, Denise Currie, Heike Schroder, Jermaine Ravalier, Patricia Nicholl, Daniel McFadden, Jana Ross

**Affiliations:** 1 School of Applied Social and Policy Sciences, Magee Campus, Ulster University, Londonderry BT48 7JL, UK; r.neill@ulster.ac.uk (R.D.N.); p.nicholl2@ulster.ac.uk (P.N.); d.mcfadden@ulster.ac.uk (D.M.); j.ross@ulster.ac.uk (J.R.); 2 School of Social Sciences, Education and Social Work, Queen’s University Belfast, 69–71 University Street, Belfast BT7 1HL, UK; j.moriarty@qub.ac.uk; 3 School of Nursing, Jordanstown Campus, Ulster University, Shore Road, Newtownabbey BT37 0QB, UK; p.gillen@ulster.ac.uk; 4 Southern Health and Social Care Trust, 10 Moyallen Road, Gilford BT63, UK; 5 School of Psychology, Coleraine Campus, Ulster University, Cromore Road, Coleraine BT52 1SA, UK; j.mallett@ulster.ac.uk; 6 NIHR Health and Social Care Workforce Research Unit, King’s College London, London, 22 Kingsway, Holborn, London WC2B 6LE, UK; jill.manthorpe@kcl.ac.uk; 7 Queen’s Management School, Queen’s University Belfast, Riddel Hall, 185 Stranmillis Road, Belfast BT9 5EE, UK; d.currie@qub.ac.uk (D.C.); h.schroder@qub.ac.uk (H.S.); 8 School of Science, Bath Spa University, Newton Park, Newton St Loe, Bath BA2 9BN, UK; j.ravalier@bathspa.ac.uk

**Keywords:** COVID-19, healthcare workforce, care workforce, United Kingdom, coping, wellbeing, quality of working life, survey

## Abstract

As the COVID-19 pandemic continues to evolve around the world, it is important to examine its effect on societies and individuals, including health and social care (HSC) professionals. The aim of this study was to compare cross-sectional data collected from HSC staff in the UK at two time points during the COVID-19 pandemic: Phase 1 (May–July 2020) and Phase 2 (November 2020–January 2021). The HSC staff surveyed consisted of nurses, midwives, allied health professionals, social care workers and social workers from across the UK (England, Wales, Scotland, Northern Ireland). Multiple regressions were used to examine the effects of different coping strategies and demographic and work-related variables on participants’ wellbeing and quality of working life to see how and if the predictors changed over time. An additional multiple regression was used to directly examine the effects of time (Phase 1 vs. Phase 2) on the outcome variables. Findings suggested that both wellbeing and quality of working life deteriorated from Phase 1 to Phase 2. The results have the potential to inform interventions for HSC staff during future waves of the COVID-19 pandemic, other infectious outbreaks or even other circumstances putting long-term pressures on HSC systems.

## 1. Introduction

The outbreak of coronavirus (COVID-19) created a worldwide pandemic following its first detection in Wuhan, China, in December 2019. Since then, 143,589,411 confirmed cases and 3,058,632 deaths (21 April 2021) have been reported to the World Health Organisation (WHO) across 219 countries and territories [1,2]. In the United Kingdom (UK) alone, 4,393,307 cases of COVID-19 and 150,841 deaths have been reported as of 20 April 2021 [3]. Over the last year, COVID-19 has had a profound impact at several levels: educational, political, societal, environmental, economic and specifically within the health and social care sector. While considerable efforts are being made, such as lockdowns, social distancing, good hygiene and vaccination programmes promoted through mainstream and social media, the world will continue to be negatively impacted [4,5,6]. Indeed, this pandemic not only continues to impact health and social care services but is still affecting the mental wellbeing and quality of life across the population, which may have a long-lasting and detrimental effect [6,7,8,9,10,11,12,13,14,15].

While everyone has been affected by COVID-19, this challenging period has taken a disproportionate toll on the health and social care workforce [12,13,16,17,18,19,20,21]. Prior to the COVID-19 crisis, UK health and social care professionals were already categorised as a high-risk group for developing mental health- and wellbeing-related problems [16,22,23,24,25,26,27]. Therefore, as the pandemic developed, this increased the job demands, burnout and stressors of the health and social care profession [15,22,23,24,28,29,30,31,32].

Protecting the mental health and wellbeing of health and social care professionals is necessary for the long-term sustainability and capacity of the workforce [33]. Currently, vaccines are being readily distributed across the UK with the hope that this should reduce the demands within the health and social care system. However, COVID-19 may have lasting effects on the workforce without increased support, clear policies and the implementation of key coping strategies or interventions to assist. The pandemic has magnified the challenges within the health and social care system while further increasing the demands on the workforce, with severe implications in terms of mental wellbeing, health, coping and work-related quality of life [15,26,28,32,34].

Previous research with healthcare professionals during the SARS (Severe Acute Respiratory Syndrome) and MERS (Middle East Respiratory Syndrome) epidemics highlighted that, even after these outbreaks eased, individuals were still mentally and physically distressed, with increased concerns for their health, job, work–life balance and family [15,35,36,37,38,39,40,41]. Lee et al. [38] highlighted that, a year after the 2003 SARS outbreak, healthcare workers still had higher levels of psychological stress, depression and anxiety in comparison to the non-healthcare workforce. Often, the rationale behind this is that health and social care professionals deal with more unusual stressors and challenges during such outbreaks or epidemics [27,35,37,39].

In addition to these findings, several recent studies and reviews have demonstrated that health and social care professionals are at a significant risk of developing severe mental health problems due to the COVID-19 pandemic [16,18,34,42,43,44,45]. Cai et al. [44] found that frontline healthcare workers had more mental health-related problems than those not on the frontline; however, their study demonstrated similar coping and treatment strategies for these individuals. Similar findings have been reported in multiple studies, indicating a significant psychological impact on the health and social care workforce during the COVID-19 pandemic [45,46,47]. Results from the Northern Ireland Health and Social Care Trust survey conducted by Shannon et al. [46] and a study by Siddquari et al. [48] reported that anxiety and depression in health and social care professionals were higher than in comparison to that reported in China [11] or the general UK population [47].

These findings have demonstrated that when the COVID-19 pandemic begins to decrease, several health and social care professionals will have suffered a psychological impact from their work and its constantly changing high pressure demands. Therefore, Blake et al. [17] and Levin [35] argued that we need to understand how this workforce is affected and, in turn, how this burden can be reduced and a sufficient level of coping achieved. While several studies have indicated that coping mechanisms are being used by health and social care staff, such as problem-focused measures, emotional-focused measures, self-rescue, positive attitudes, refocusing, appraisal mechanisms and social support from loved ones, more help with coping may be required to improve or sustain mental health and wellbeing [19,27,44,45,49,50].

### Aims of the Study

Data exploring a cross-sectional examination of mental wellbeing and quality of working life in health and social care workers in the UK during the COVID-19 pandemic is currently lacking. It is possible that mental wellbeing and quality of working life will continue to worsen across the UK health and social care workforce between the start of the pandemic and the present time (April 2021). Accordingly, the purpose of the current study is to expand on the evidence base relating to the impact of COVID-19 on the health and social care workforce. 

The current study aimed to compare cross-sectional data collected from health and social care professionals in the UK at two different time points (Phase 1: May–July 2020; Phase 2: Nov 2020–Jan 2021) during the COVID-19 pandemic. It is important to investigate the influence of job-related factors on workforce wellbeing, during the pandemic, specifically, to examine coping strategies, mental wellbeing and quality of working life. This paper provides insights into the impact of the pandemic over time for a range of health and social care workers and explores the significance of certain variables between Phase 1 and Phase 2. Examining the difference in wellbeing and work-related quality of life over the course of the pandemic is crucial in helping to identify any relevant supports for this workforce. The results of this study will be used to inform policies and procedures for health and social care employers to mitigate the adverse long-term effects on mental wellbeing and work-related quality of life.

## 2. Materials and Methods

### 2.1. Design and Participants

This study forms a part of an ongoing research programme entitled ‘Health and social care workers’ quality of working life and coping while working during the COVID-19 pandemic’. The research explores the impact of providing health and social care during the COVID-19 pandemic on nurses, midwives, allied health professionals (AHPs), social care workers and social workers in the UK working in a range of settings such as hospitals, care homes (including nursing homes), community and day services. The wider study uses a cross-sectional design, and the data for the current study presented here were collected at two time points: Phase 1 ran from 7 May to 3 July 2020, and Phase 2 ran from 17 November 2020 to 1 February 2021. At both times, the data were collected anonymously online through Qualtrics. Phase 1 received a total of 3290 responses and Phase 2 received 3499 responses. 

Participation in the study at each time point was voluntary, with participants recruited by a convenience sampling method. The number of nurses and midwives in the UK is 660,213 and 37,255, respectively [51]. Using the Raosoft sample calculator [52] with a confidence interval of 95%, the sample that we hoped to recruit for each phase was 384 nurses and 381 midwives. The number of AHPs working in health and social care in the UK is 152,000 [53]; therefore, using the Raosoft sample calculator [53], it was aimed to recruit 384. The number of social care workers and social workers in Northern Ireland is 37,779 and 6357, respectively. Using the Raosoft sample calculator with a confidence interval of 95%, the sample that we hoped to recruit contained 381 social care workers and 363 social workers.

Participants recruited included nurses, midwives, AHPs, social care workers and social workers in the UK who had signed up to receive newsletters or journals from professional associations, workplace unions and regulators such as the Royal College of Nursing (RCN), Royal College of Midwives (RCM), the Northern Ireland Practice and Education Council (NIPEC), Northern Ireland Social Care Council (NISCC), the Royal College of Occupational Therapists, British Dietetic Association and others. Additionally, in order to reach a wider population of health and social care workers in the UK, social media platforms such as Twitter and Facebook were used to advertise the survey, with an electronic link and QR code to the participant information sheet, consent and survey. Study eligibility was based on participants self-reporting their occupation as a nurse, midwife, AHP, social care worker or social worker and working either in England, Scotland, Wales or Northern Ireland. Demographic and work-related characteristics of the effective sample by study phase are presented in Table 1.

### 2.2. Ethical Considerations

Ethical approval was obtained from the Research Ethics Filter Committee of the School of Nursing at Ulster University (Ref No: 2020/5/3.1, 23 April 2020; Ulster University IRAS Ref No: 20/0073) for Phases 1 and 2. Trust Governance approval was gained from the Health and Social Care (HSC) Trusts in Norterhn Irelandfor Phase 2, which allowed the link to the anonymised questionnaire to be shared with HSC staff via Trust emails. Permission to use the scales in the questionnaire was provided by the original authors of the scales, and consent, confidentiality and anonymity were addressed in participant information sheets prior to the commencement of the survey.

### 2.3. Measures

#### 2.3.1. Demographics and Work-Related Characteristics

As part of the wider questionnaire, participants were asked about their demographic and work-related characteristics. The variables that were consistent across Phase 1 and Phase 2 of the wider study and are relevant to the current analyses are sex, age, ethnicity, country of work, occupational group, disability and years of experience. The response options were presented as categories, and, in the current study, some of these were collapsed to account for small sample sizes in certain sub-groups.

#### 2.3.2. Mental Wellbeing

Mental wellbeing was assessed with the Short Warwick Edinburgh Mental Wellbeing Scale (SWEMWBS) [54], a seven-item scale enquiring about participants’ feelings and thoughts over the last two weeks. A five-point Likert scale (1 = ‘None of the time’ to 5 = ‘All of the time’) is used to rate the items. The scores for the individual items were summed and transformed into metric scores [54]. Total scores can range from 7 to 35, with higher scores indicating better mental wellbeing. Cronbach’s alpha for the seven items was acceptable in the current study (Phase 1: α = 0.862; Phase 2: α = 0.862).

#### 2.3.3. Quality of Working Life

Quality of working life was assessed through the Work-Related Quality of Life (WRQOL) [55] scale consisting of 24 items. Twenty-three of these contribute to the final score (not including the 24th ‘overall’ item) and are rated on a five-point Likert scale (1 = ‘Strongly disagree’ to 5 = ‘Strongly agree’). Scores can range from 23 to 115 and higher scores indicate better overall quality of working life. Cronbach’s alpha for the 23 items was acceptable in the current study (Phase 1: α = 0.878; Phase 2: α = 0.885). 

#### 2.3.4. Coping

The way in which participants claimed that they coped during the pandemic was assessed using items from two different scales. First, 20 items corresponding to ten different coping strategies (active coping, planning, positive reframing, acceptance, use of emotional support, use of instrumental support, venting, substance use, behavioural disengagement, self-blame) from the Brief COPE scale [56] were used. Participants were asked to indicate how often they had been doing what was described by the 20 statements in relation to their coping with COVID-19-related occupational demands at the present time. A four-point Likert scale (1 = ‘I haven’t been doing this at all’ to 4 = ‘I’ve been doing this a lot’) was used to record the responses. Each coping strategy was assessed with two items, which were summed, and higher scores indicated that participants used that particular coping strategy more often. Cronbach’s alpha for the 20 items was acceptable (Phase 1: α = 0.802; Phase 2: α = 0.831).

Additionally, participants were asked to complete 15 items from Clark, Michel, Early and Baltes’ [57] scale, which assessed five different coping strategies (family–work segmentation, work–family segmentation, working to improve skills/efficiency, recreation and relaxation, exercise). These coping strategies were selected to supplement the Brief COPE domains. Participants used a six-point Likert scale (1 = ‘Never have done this’ to 6 = ‘Almost always do this’) to indicate how often they personally do what is described in the items to cope with work stressors. The five coping strategies are represented by three items each and a mean score for each strategy is computed. Higher scores indicate greater frequency of use of that particular coping strategy. Cronbach’s alpha for the 15 items used was acceptable (Phase 1: α = 0.832; Phase 2: α = 0.829).

### 2.4. Data Analysis

All analyses were conducted in SPSS 26 and Mplus 7.3. Missing data were addressed prior to the analyses. Initially, participants who did not complete any items on one or more of the scales (SWEMWBS, WRQOL, Brief COPE, Clark’s coping) were excluded (*n* = 1466), leaving an effective sample of 5323 participants (2555 from Phase 1 and 2768 from Phase 2). The remaining missing data on the variables relevant to the analyses were 0.30%. The SWEMWBS, WRQOL and the coping items were treated as continuous variables and missing data on these items were estimated using the EM algorithm in SPSS. Missing values on the demographic and work-related variables were minimal and they were not estimated. Instead, listwise deletion was used in the regression analyses.

A multivariate multiple regression was conducted to examine the association between the study phase and participants’ wellbeing and quality of working life. In the first step, the analysis controlled for the effects of participants’ demographic and work-related characteristics (sex, age, ethnic background, country of work, occupational group, number of years of work experience, disability status). In the second step, coping strategies were added to the model as covariates (10 Brief COPE strategies and five Clark’s coping strategies).

A multivariate multiple group regression was then conducted with the coping strategies as predictor variables, wellbeing and quality of working life as outcome variables, demographic and work-related characteristics as covariates and study phase as a moderating variable. This model was run as a multiple group analysis to enable comparison of the effects across study phases. Wald tests of parameter estimates were used to compare the effects of the coping strategies on the outcome variables across the two study phases. A significant Wald test indicates that the difference between two parameters is significantly different from zero.

In order to account for the different distribution of occupational groups and countries across the two study phases, descriptive statistics for the outcome variables (wellbeing, quality of working life) and the coping strategies were weighted by occupation and country. Frequencies and percentages describing the two samples and the regression analyses were unweighted.

## 3. Results

### 3.1. Prelimary Analyses

Descriptive statistics for Phase 1 and Phase 2 of the study, including simple comparisons across phases, are presented in Table 2. The results show that wellbeing and quality of working life were lower in Phase 2 compared to Phase 1 of the study. Participants also appeared to be using positive coping strategies (e.g., active coping, positive reframing, acceptance) less frequently and negative coping strategies (e.g., venting, behavioural disengagement, self-blame) more frequently in Phase 2 compared to Phase 1 of the study.

### 3.2. Regression Analyses

The regression analysis examining the association between the study phase and participants’ wellbeing, whilst controlling for demographic and work-related characteristics, revealed a significant effect of the study phase, such that wellbeing was significantly higher in Phase 1 compared to Phase 2 (b = 0.887, β = 0.252, *p* < 0.001; R^2^ = 0.058, SE = 0.006, *p* < 0.001). Similar results were found for quality of working life, which also deteriorated from Phase 1 to Phase 2 (b = 3.527, β = 0.228, *p* < 0.001; R^2^ = 0.057, SE = 0.006, *p* < 0.001). Both models, however, explained very little variance in the outcomes.

When coping strategies were added to the model, the effects of study phase on the outcome variables disappeared, suggesting no significant differences in wellbeing and quality of working life between Phase 1 and Phase 2 of the study (Wellbeing: b = −0.057, β = −0.008, *p* = 0.495; Quality of working life: b = 0.111, β = 0.004, *p* = 0.784), and the models explained substantially more variance (Wellbeing: R^2^ = 0.406, SE = 0.012, *p* < 0.001; Quality of working life: R^2^ = 0.319, SE = 0.012, *p* < 0.001).

The effects of coping strategies on wellbeing and quality of working life in Phase 1 and Phase 2 of the study are presented in Table 3 and Table 4, respectively, along with a comparison of their effects on the outcomes. As shown in the two tables, almost all coping strategies were significantly associated with the outcomes in both study phases, some positively (e.g., active coping, positive reframing, acceptance) and others negatively (e.g., planning, venting, substance use). There was very little variation in the strength of the associations, as shown by Wald tests of parameter constraints; in relation to wellbeing, active coping was more strongly (and positively) associated with wellbeing in Phase 1, use of emotional support was more strongly (and positively) associated with wellbeing in Phase 2, and family–work segmentation was more strongly (and negatively) associated with wellbeing in Phase 2. In relation to the quality of working life, use of emotional support was more strongly (and positively) associated with quality of working life in Phase 2, and behavioural disengagement was more strongly (and negatively) associated with quality of working life in Phase 2.

## 4. Discussion

### 4.1. Summary of Findings and Comparison with Other Literature

The current study compared cross-sectional data collected from health and social care staff in the UK at two time points during the COVID-19 pandemic: Phase 1 (May–July 2020) and Phase 2 (November 2020–January 2021). The results indicated that when controlling for demographic and work-related characteristics, both wellbeing and WRQOL deteriorated from Phase 1 to Phase 2 of the study. However, once coping strategies were added to the model and controlled for, this effect of study phase disappeared and the differences in wellbeing and WRQOL were no longer significant between the two phases of the study. This suggests that coping strategies played an important role in the health and social care workers’ wellbeing and WRQOL as the pandemic progressed. Furthermore, as restrictions/lockdown continued and COVID-19 cases rose across the UK, the findings suggest that changes could be a result of different coping strategies, particularly as the findings demonstrated that the type of strategy utilised changed over the course of the pandemic. Evidence supporting this finding indicates that coping strategies during and after crises such as COVID-19 can help to reduce the burden of increased stressors associated with this pandemic [16,17,25,35,50,58,59,60,61]. Coping strategies are intended to reduce stressors and regulate emotions, thus lowering levels of anxiety and improving mental wellbeing [61,62]. These behaviours can help people to learn to deal with stress in unprecedented and challenging situations such as the COVID-19 pandemic and assist in reducing negative thoughts, emotions and behaviours.

Previous research has demonstrated that stress and coping strategies are related [37,61,63,64]. Evidence indicates that the psychological impact and stress of COVID-19 on health and social care workers will exacerbate poor mental wellbeing alongside increased anxiety and depression [12,13,60,65]. The current study found that the coping strategies that were significantly associated with wellbeing and WRQOL in Phase 1 of the study were also significantly associated with these outcomes in Phase 2 of the study. Additionally, the effects of these coping strategies on the outcomes remained largely unchanged from Phase 1 to Phase 2, suggesting that strategies that were important for better wellbeing and WRQOL in Phase 1 continued to be important in Phase 2. In the same vein, strategies that contributed to worse wellbeing and WRQOL in Phase 1 also contributed to worse wellbeing and WRQOL in Phase 2. Almost all coping strategies were significantly associated with the outcomes in both study phases, some positively (e.g., active coping, positive reframing, acceptance) and others negatively (e.g., planning, venting, avoidance, social isolation, substance use).

A survey conducted by Man et al. [45] in Romania examined disease perception and coping with emotional distress in healthcare staff during COVID-19. These authors highlighted that coping mechanisms such as positive appraisal, reframing and social support of loved ones were used more by healthcare staff than across the general population. Similar findings were outlined within this present study as participants used similar positive coping strategies (e.g., active coping, emotional support, positive reframing, acceptance). This further demonstrates that individuals who work in the health and social care sector are more under pressure at times and require coping strategies to deal with stressors daily. Additionally, researchers have suggested that social support and active coping are particularly important coping strategies which are often found to have a positive effect on wellbeing and quality of working life [16,44,49,60,66,67,68]. These findings support this present study, which suggests that social support is associated positively with wellbeing.

An important finding of this present study was that behavioural disengagement was found to be negatively associated with quality of working life. This suggests that as the pandemic continues, the increased stressors associated with the health and care sector may lead to further deterioration of wellbeing and work-related quality of life. Similarly, studies by Flesia et al. [60] and Babore et al. [65] also found that negative coping strategies such as higher levels of avoidance negatively impacted psychological state. Emotion-focused coping appears to have a negative adverse effect on mental health and mental wellbeing [19,27,44,62,69,70].

While there was a decrease in the use of positive coping strategies and an increase in the use of the negative coping strategies highlighted within this present study, the effects of the coping strategies on the outcome variables remained largely unchanged. This indicates that the decrease in wellbeing and WRQOL may be due to people not coping so efficiently in the later stages of the pandemic. Evidence suggests that as the COVID-19 pandemic further unfolds, it will continue to take a heavy toll on healthcare workers as their ability to cope becomes more affected due to increased uncertainty and job demands in these unprecedented times [8,16,18,19,58,71,72,73]. This is perhaps a further explanation of why wellbeing and WRQoL deteriorated between Phases 1 and 2 of this study.

Another explanation for this deterioration is that there may not be enough social support across the organisational levels in the workplace. This, combined with work tensions, can lead to increased stressors and burnout, which can have a negative influence on wellbeing and work-related quality of life [28,68,74,75,76,77]. Stress response can lead to further deterioration [74]. This becomes more problematic as several coping mechanisms may become unsuccessful, which may lead to an appraisal of the stressors that are mentally more threatening and uncontrollable [61,78].

Overall, the findings suggest that coping strategies are important for wellbeing and WRQoL, indicating that coping skills training might be helpful for the health and social care workforce during this pandemic. Blake et al. [17] acknowledged that establishing such programmes might help mitigate the psychological impact of COVID-19 on the health and social care workforce. Balasubramanian et al. [79] also indicated that capitalising on positive coping behaviours is one way to stem the deterioration and therefore coping skills are essential to develop. Research suggests that training and communication to improve one’s ability to cope prior to disasters or crises may be needed, particularly as staff wish for better coping strategies such as self-rescue and positivity [49,62,72,73,80]. Different strategies can be adopted or combined to create the most effective coping methods. However, it must be acknowledged that coping responses will differ due to individual preference and cognitive state therefore more than one plan is required [61,63,72]. Additionally, the findings suggest that there is no best strategy to implement as many coping techniques are effective, therefore, individual choice will be important and how effective such techniques are will depend on whether a positive strategy is used efficiently. 

### 4.2. Limitations and Strengths

The main advantage of this study is its comparison of wellbeing, quality of working life and coping over two different time points during the COVID-19 pandemic. Given the fluctuating nature of the pandemic, this study is important in capturing the difference in wellbeing and work-related quality of life over the course of the pandemic, which is crucial in helping to identify the challenges related to this workforce. This is a strength of the cross-sectional design, which allows for associations across multiple outcomes to be examined to understand prevalence [81]. Additionally, the inclusion of a range of health and social care professionals (nurses, midwives, AHPs, social care workers and social workers) with a wide range of experience in the study was a strength as it provided evidence from multiple settings and circumstances.

The current study has several limitations that should be considered, and it is important to note that the data are not representative of the general UK population. Firstly, data were collected through an online survey, which could only be accessed by individuals who had seen the recruitment advertisement for the study, and specific groups may have been underrepresented due to their lack of social media usage, online platforms, technological devices or internet access. Therefore, these findings may not be generalisable to the wider UK population as a whole and may be at risk of selection bias [82,83,84]. Secondly, the study involved cross-sectional data, meaning that it is only reflective of that single point of time for participants; therefore, we cannot infer cause and effect [81]. Conversely, whilst this is a limitation, the strength of the study is the timeliness of data collection in relation to the pandemic, at time points which reflect different levels of pressures for the workforce. Another strength is the number of responses received at both study phases. 

Thirdly, over 75 percent of the sample were part of the social work and social care professions and therefore not representative of the whole health and social care sector. This makes the findings of this study less generalisable to the health workforce in these countries. The research team mitigated these limitations by weighting the data during the statistical analysis, which diminishes the effects of inherent biases of the survey and allows for a more accurate representation of the population being examined. Additionally, the over-representation of females in the sample (87%) may have influenced the results of the research and thus generalisations to their male counterparts must be considered tentatively. However, this gender composition is similar to previous research which involves a larger female participant sample in comparison to males [11,21,47,85,86] and is representative of the composition of the workforce, as females account for 70 percent of the surveyed workforces [42,71]. Finally, the self-reported nature of the survey data opens up the possibility that some participants may have provided answers that were subject to risks of social desirability bias or recall bias [87,88,89]. 

### 4.3. Implications

This study has important implications for the health and social care workforce, managers and policymakers during and after the COVID-19 pandemic. Protecting the wellbeing and improving the quality of working life are important; however, this can only be achieved through appropriate measures taken in a timely manner. Findings from this study echo previous epidemic contexts where a small number of studies examined outbreaks and their effect on health and social care professionals. This highlights the necessity for health and social care systems to address psychological wellbeing and quality of working life with their workforce across many different levels. Additional support and services are needed to prevent further deterioration of these outcomes and create future improvements for this workforce.

The findings of this study highlight the use of positive coping techniques including active coping and emotional support that were common and beneficial across the workforce. However, behavioural disengagement and family–work segmentation were negative factors. This suggests the importance of coping strategies and indicates that support needs to be in place across multiple system levels (individual, organisational and policy) to help the workforce to recover from the demands and increased stressors that the pandemic has caused. Staff wellbeing is important, and, during this pandemic, many have been in a prolonged period of unprecedented stress and pressure. The pandemic has highlighted that health and social care staff are already at risk of mental health-related problems due to their demanding schedules, responsibilities and a lack of resources. Therefore, concerned efforts are required to make work within this sector less burdensome and a more attractive employment option by examining pay and work conditions and allowing the workforce to have a voice, with the ability to raise any concerns in a supportive environment. 

Wellbeing services should be further developed as staff have demonstrated their appreciation of these programmes, while training for redeployment and skill development would be beneficial. Evidence has suggested that training and preparation are essential strategies alongside consideration of personalised plans [49,59,72,73,80]. Managers need to ensure that staff can take annual leave and regular breaks as this will provide them with an opportunity to de-stress. Communication will also be an important practice at both individual and organisational level, with staff being signposted as needed to support services for counselling and mentoring support. The ability to sustain and create longer-term programmes is vital in continuing to improve wellbeing and work-related quality of life post-pandemic [74]. Additionally, creating a positive team culture and support system across all employees will also be beneficial.

These strategies and programmes may be challenging to implement, yet they will help to improve resilience and wellbeing, which can help to tackle staff burnout and improve work-related quality of life. Additionally, by introducing these strategies across multiple levels, it can help to improve the workforce’s capacity to deal with any possible future epidemic or pandemic situations. If we are not proactive in establishing training or coping programmes for this workforce, there may continue to be a deterioration in wellbeing and quality of work-related life. Future examination of coping through qualitative studies is warranted to further understand how coping mechanisms can be developed and implemented further to improve wellbeing and work-related quality of life in the health and social care workforce.

## 5. Conclusions

The COVID-19 pandemic continues to present a psychological impact on mental wellbeing and quality of working life for the health and social care workforce. The present study provided insight into the cross-sectional data collected from such staff in the UK at two time points during the COVID-19 pandemic: Phase 1 (May–July 2020) and Phase 2 (November 2020–January 2021). Importantly, this study demonstrates that both wellbeing and quality of working life deteriorated between Phase 1 and Phase 2. The results have the potential to inform interventions, training and coping strategies for staff during future waves of the COVID-19 pandemic, other infectious outbreaks or other circumstances putting long-term pressures on the health and care systems. The findings suggest that employee support across multiple levels must be deployed to establish good communication, connections and conditions to support staff’s wellbeing and quality of working life.

## Figures and Tables

**Table 1:** Demographic and work-related characteristics of the effective sample (Phase 1: *n* = 2555; Phase 2: *n* = 2768).

Variable	Phase 1 (7 May–3 July 2020)	Phase 2 (17 November 2020–1 February 2021)
**Sex**		
Female	2221 (87.23%)	2441 (88.25%)
Male	325 (12.77%)	325 (11.75%)
**Age**		
16–29	306 (11.98%)	307 (11.09%)
30–39	541 (21.18%)	640 (23.12%)
40–49	755 (29.56%)	729 (26.34%)
50–59	757 (29.64%)	827 (29.88%)
60–65	178 (6.97%)	230 (8.31%)
66+	17 (0.67%)	35 (1.26%)
**Ethnic background**		
White	2402 (94.16%)	2655 (96.09%)
Black	74 (2.90%)	40 (1.45%)
Asian	29 (1.14%)	26 (0.94%)
Mixed	46 (1.80%)	42 (1.52%)
**Country of work**		
England	910 (35.62%)	642 (23.19%)
Scotland	107 (4.19%)	358 (12.93%)
Wales	147 (5.75%)	856 (30.92%)
Northern Ireland	1391 (54.44%)	912 (32.95%)
**Occupational group**		
Nursing	142 (5.56%)	291 (10.51%)
Midwifery	139 (5.44%)	59 (2.13%)
Allied Health Professionals	312 (12.21%)	500 (18.06%)
Social Care	922 (36.09%)	961 (34.72%)
Social Work	1040 (40.70%)	957 (34.57%)
**Number of years of work experience**		
Less than 2 years	211 (8.26%)	184 (6.65%)
2–5 years	377 (14.76%)	379 (13.70%)
6–10 years	407 (15.93%)	454 (16.41%)
11–20 years	688 (26.93%)	842 (30.43%)
21–30 years	575 (22.50%)	558 (20.17%)
More than 30 years	297 (11.62%)	350 (12.65%)
**Disability status**		
Yes	225 (8.81%)	275 (9.94%)
No	2273 (88.96%)	2430 (87.82%)
Unsure	57 (2.23%)	62 (2.24%)

Note. Presented are column percentages, which are valid percentages to account for missing data.

**Table 2:** Descriptive statistics for key study variables and their comparison between Phase 1 and Phase 2 of the study.

Variable	Unweighted Results			Weighted Results ^1^		
	Phase 1 (*n =* 2555)	Phase 2 (*n =* 2768)	Measured Change	Phase 1 (*n =* 2555)	Phase 2 (*n =* 2768)	Measured Change
	M (SD)		Mean Difference	M (SD)		Mean Difference
Wellbeing	21.35 (3.59)	20.43 (3.40)	0.92 **	20.94 (3.79)	20.21 (3.36)	0.73 **
Quality of working life	78.14 (15.32)	75.64 (15.59)	2.50 **	78.03 (17.51)	72.56 (15.93)	5.47 **
*Coping strategies*						
Active coping	6.08 (1.61)	5.51 (1.68)	0.57 **	6.00 (1.64)	5.48 (1.71)	0.51 **
Planning	5.84 (1.75)	5.48 (1.83)	0.36 **	5.81 (1.81)	5.52 (1.86)	0.29 **
Positive reframing	5.96 (1.62)	5.64 (1.67)	0.31 **	5.85 (1.65)	5.57 (1.70)	0.28 **
Acceptance	6.49 (1.43)	6.18 (1.53)	0.31 **	6.39 (1.53)	6.18 (1.51)	0.21 **
Use of emotional support	5.01 (1.77)	4.91 (1.79)	0.10	4.93 (1.76)	4.74 (1.83)	0.19 **
Use of instrumental support	4.47 (1.73)	4.50 (1.76)	−0.03	4.34 (1.83)	4.29 (1.79)	0.05
Venting	3.52 (1.40)	4.22 (1.64)	−0.69 **	3.51 (1.43)	4.15 (1.64)	−0.64 **
Substance use	2.78 (1.40)	2.89 (1.49)	−0.11 *	2.76 (1.41)	2.81 (1.44)	−0.05
Behavioural disengagement	2.62 (1.19)	2.96 (1.39)	−0.33 **	2.73 (1.26)	3.00 (1.38)	−0.27 **
Self-blame	3.27 (1.60)	3.93 (1.83)	−0.65 **	3.42 (1.80)	4.01 (1.87)	−0.59 **
Family–work segmentation	5.05 (0.91)	5.06 (0.92)	−0.01	5.14 (0.84)	5.12 (0.84)	0.02
Work–family segmentation	4.71 (1.06)	4.60 (1.10)	0.11 **	4.67 (1.06)	4.59 (1.07)	0.08 *
Working to improve skills/efficiency	4.33 (1.05)	4.22 (1.09)	0.11 **	4.49 (1.09)	4.18 (1.15)	0.31 **
Recreation and relaxation	3.76 (1.22)	3.59 (1.25)	0.17 **	3.75 (1.23)	3.55 (1.31)	0.20 **
Exercise	3.93 (1.35)	3.71 (1.41)	0.22 *	3.97 (1.42)	3.65 (1.38)	0.32 **

Note. ^1^ The results were weighted by country of work and occupational group * *p* < 0.005, ** *p* < 0.001.

**Table 3:** Multiple group regression analysis examining coping strategies as predictors of wellbeing.

Predictor Variable	Wellbeing				
	Phase 1 (*n =* 2541)		Phase 2 (*n =* 2759)		Phase 1 vs. Phase 2 Comparison (*n =* 5300)
	b	β	b	β	*p*-Value
Active coping	0.324 **	0.146 **	0.118 *	0.058 *	0.005
Planning	−0.192 **	−0.094 **	−0.102 *	−0.055 *	0.192
Positive reframing	0.283 **	0.128 **	0.171 **	0.084 **	0.096
Acceptance	0.198 **	0.079 **	0.251 **	0.113 **	0.430
Use of emotional support	0.254 **	0.126 **	0.375 **	0.198 **	0.038
Use of instrumental support	−0.040	−0.019	−0.031	−0.016 (0.432)	0.880
Venting	−0.230 **	−0.090 **	−0.166 **	−0.080 **	0.288
Substance use	−0.131 **	−0.051 *	−0.087 *	−0.038 *	0.412
Behavioural disengagement	−0.265 **	−0.087 **	−0.289 **	−0.118 **	0.734
Self-blame	−0.603 **	−0.270 *	−0.529 **	−0.285 **	0.154
Family–work segmentation	−0.084	−0.021	−0.296 **	−0.080 **	0.044
Work–family segmentation	0.140 *	0.042 *	0.157 *	0.050 *	0.848
Working to improve skills/efficiency	0.334 **	0.098 **	0.362 **	0.116 **	0.745
Recreation and relaxation	0.154 *	0.052 *	0.189 **	0.069 **	0.670
Exercise	0.152 *	0.057 *	0.105 *	0.044 *	0.476

Note. b = unstandardised estimate; β = standardised estimate. All analyses controlled for participants’ sex, age, ethnic background, country of work, occupational group, number of years of work experience and disability status (* *p* < 0.005, ** *p* < 0.001).

**Table 4:** Multiple group regression analysis examining coping strategies as predictors of quality of working life.

Predictor Variable	Quality of Working Life				
	Phase 1 (*n =* 2541)		Phase 2 (*n =* 2759)		Phase 1 vs. Phase 2 Comparison (*n =* 5300)
	b	β	b	β	*p*-Value
Active coping	1.040 **	0.110 **	0.390	0.042	0.059
Planning	−1.097 **	−0.126 **	−0.899 **	−0.105 **	0.533
Positive reframing	1.141 **	0.121 **	0.711 *	0.076 *	0.161
Acceptance	0.168	0.016	0.669 *	0.065 *	0.111
Use of emotional support	1.172 **	0.136 **	1.718 **	0.198 **	0.045*
Use of instrumental support	−0.156	−0.018	−0.387 *	−0.044 *	0.419
Venting	−1.237 **	−0.113 **	−0.784 **	−0.083 **	0.106
Substance use	−0.218	−0.020	−0.061	−0.006	0.564
Behavioural disengagement	−0.827 *	−0.064 *	−1.574 **	−0.140 **	0.041 *
Self-blame	−1.995 **	−0.209 **	−1.517 **	−0.179 **	0.078
Family–work segmentation	−1.368 **	−0.081 **	−2.243 **	−0.133 **	0.062
Work–family segmentation	1.104 *	0.077 *	1.795 **	0.126 **	0.107
Working to improve skills/efficiency	1.896 **	0.130 **	1.982 **	0.139 **	0.832
Recreation and relaxation	1.047 **	0.083 **	1.071 **	0.086 **	0.948
Exercise	−0.124	−0.011	0.088	0.008	0.485

Note. b = unstandardised estimate; β = standardised estimate. All analyses controlled for participants’ sex, age, ethnic background, country of work, occupational group, number of years of work experience and disability status (* *p* < 0.005 ** *p* < 0.001).

## Data Availability

The data presented in this study are available on request from the corresponding author. The data are not publicly available due to ethical restrictions.
